# How Do European Pharmacy Students Rank Competences for Practice?

**DOI:** 10.3390/pharmacy4010008

**Published:** 2016-01-26

**Authors:** Jeffrey Atkinson, Kristien De Paepe, Antonio Sánchez Pozo, Dimitrios Rekkas, Daisy Volmer, Jouni Hirvonen, Borut Bozic, Agnieska Skowron, Constantin Mircioiu, Annie Marcincal, Andries Koster, Keith Wilson, Chris van Schravendijk, Sandra Hočevar

**Affiliations:** 1 Pharmacology Department, Lorraine University, 5 rue Albert Lebrun, 54000 Nancy, France; 2 Pharmacolor Consultants Nancy, 12 rue de Versigny, 54600 Villers, France; 3 Pharmacy Faculty, Vrije Universiteit Brussel, Laarbeeklaan 103, Brussels 1090, Belgium; kdepaepe@vub.ac.be; 4 Faculty of Pharmacy, University of Granada, Campus Universitario de la Cartuja s/n, Granada 18701, Spain; sanchezpster@ugr.com; 5 School of Pharmacy, National and Kapodistrian University Athens, Panepistimiou 30, Athens 10679, Greece; rekkas@pharm.uoa.gr; 6 Pharmacy Faculty, University of Tartu, Nooruse 1, Tartu 50411, Estonia; daisy.volmer@ut.ee; 7 Pharmacy Faculty, University of Helsinki, Yliopistonkatu 4, P.O. Box 33-4, Helsinki 00014, Finland; jouni.hirvonen@helsinki.fi; 8 Faculty of Pharmacy, University of Ljubljana, Askerceva cesta 7, Ljubljana 1000, Slovenia; Borut.Bozic@ffa.uni-lj.si; 9 Pharmacy Faculty, Jagiellonian University, Golebia 24, Krakow 31-007, Poland; askowron@cm-uj.krakow.pl; 10 Pharmacy Faculty, University of Medicine and Pharmacy “Carol Davila” Bucharest, Dionisie Lupu 37, Bucharest 020021, Romania; constantin.mircioiu@yahoo.com; 11 Faculty of Pharmacy, European Association of Faculties of Pharmacy, Université de Lille 2, Lille 59000, France; annie.marcincal@pharma.univ-lille2.fr; 12 Department of Pharmaceutical Sciences, European Association of Faculties of Pharmacy, Utrecht University, PO Box 80082, 3508 TB Utrecht, The Netherlands; A.S.Koster@uu.nl; 13 School of Life and Health Sciences, Aston University, Birmingham, B47ET, UK; k.a.wilson@aston.ac.uk; 14 Medical Faculty, Vrije Universiteit Brussel, Laarbeeklaan 103, 1090 Brussels, Belgium; chrisvs@vub.ac.be; 15 European Pharmacy Students’ Association (EPSA), Rue de Luxembourg 19/6, 1000 Brussels, Belgium; sandra.hoce@gmail.com

**Keywords:** pharmacy, education, competences, framework, student, practice

## Abstract

European students (*n* = 370), academics (*n* = 241) and community pharmacists (*n* = 258) ranked 13 clusters of 68 personal and patient care competences for pharmacy practice. The results show that ranking profiles for all three groups as a rule were similar. This was especially true of the comparison between students and community pharmacists concerning patient care competences suggesting that students have a good idea of their future profession. A comparison of first and fifth (final) year students shows more awareness of patient care competences in the final year students. Differences do exist, however, between students and community pharmacists. Students—like academics—ranked competences concerned with industrial pharmacy and the quality aspects of preparing drugs, as well as scientific fundamentals of pharmacy practice, well above the rankings of community pharmacists. There were no substantial differences amongst rankings of students from different countries although some countries have more “medicinal” courses than others. This is to our knowledge the first paper to look at how, within a healthcare sectoral profession such as pharmacy, the views on the relative importance of different competences for practice of those educating the future professionals and their students, are compared to the views of working professionals.

## 1. Introduction

The PHARMINE (Pharmacy education in Europe) [1] study aimed at promoting the use of competence frameworks in European pharmacy education. Competence frameworks have been developed to facilitate practitioner development and assessment, and have already been used in the workplace to monitor and improve practice of Singaporean hospital pharmacists [2], and of hospital pharmacists in Queensland [3]. Studies have also been conducted in the UK [4] and in Canada [5] in community pharmacy. These studies attempted to define the roles of pharmacists in a community or hospital setting and establish measurable outcomes on the possible impact of the application of competence frameworks for development and assessment. All studies concluded that competence frameworks are useful tools to monitor and improve performance in the workplace. PHARMINE and its follow-up project PHAR-QA (Quality Assurance in European Pharmacy Education and Training) [6] extended this approach to pharmacy education. A competence framework similar to that used in the four studies cited previously was used. The main difference between the two was, that with the four studies done in the workplace, the emphasis was on the fourth level of Miller’s triangle *i.e.*, “*does*” whereas in this study emphasis was one the first two levels “*knows*” and “*knows how*” (see conclusions)*.* Thus the studies in the workplace are aimed at personal development and improvement in action whereas this study is aimed more at adapting pharmacy education to a competence approach. These aspects will be taken up in the discussion following the exposé of the study and results.

The PHAR-QA (“Quality Assurance in European PHARmacy Education and Training”) project, funded by the European Commission, asked pharmacy students, academics, and community pharmacists to rank competences for pharmacy practice.

This paper asks the question of whether the ranking of competences by students is similar to that of academics and/or to that of community pharmacists. It also looks at whether their ideas on the relative ranking of competences evolve during their studies by comparing the scores of first year students with that of fifth (final) year students. Finally, it also looks at potential differences amongst students from different countries.

The methodology used is based on that in the MEDINE (“Medical Education in Europe”) project that asked medical doctors and students to rank competences for the medical practitioner [7].

## 2. Experimental Section

Ranking data on competences for practice were obtained via the PHAR-QA surveymonkey [8] questionnaire that was available online from 14 February 2014 through 1 November 2014 *i.e.*, 8.5 month [9]. Respondents came from 33/38 (Table 1) countries drawn from the European Higher Education Area [10].

**Table 1:** Student respondents by country.

The number of students who responded from each country.	
Germany	127
Czech Republic	32
Portugal	28
Romania	21
Belgium	20
Finland	18
Macedonia (FYROM)	12
Croatia	10
Malta	10
France	7
Latvia	7
Montenegro	7
Estonia	6
Greece	6
Poland	6
Slovenia	6
Spain	6
Norway	5
The Netherlands	5
Turkey	5
UK	4
Serbia	3
Switzerland	3
Austria	2
Denmark	2
Sweden	2
Ukraine	2
Albania	1
Bosnia	1
Hungary	1
Kosovo	1
Lithuania	1
Slovakia	1
Belarus	0
Bulgaria	0
Iceland	0
Ireland	0
Italy	0

Nine countries were represented by 10 or more respondents, 21 countries by four or more, five countries had no respondents. Two respondents did not answer the “country of residence” question. The questionnaire was distributed by several means. Firstly the PHAR-QA consortium nominated four regional managers (north, south, east, and west) who sent out the survey to the various countries based on their geographical location in Europe. The regional managers contacted pharmacy departments, students and organizations representing other groups, chambers, governments and other agencies. The survey was also distributed by the European Pharmacy Students’ Association via its national representatives. Thus the survey was distributed randomly and anonymously. No attempt was made to target specific groups to obtain a previously established number of respondents, as a function, for example, of the population of the country. Our approach produced an imbalanced distribution across European countries with small countries such as Malta in the top nine for respondents and large countries such as Italy having no student respondents. Furthermore no attempt was made to have an equal number of respondents for the three groups (students, academics, community pharmacists) from a given country. It was felt, however, that what the survey lost in balance it gained in being random and anonymous.

The first six questions of the survey were on the profile of the respondent asking, amongst others, country of residence, current occupation (student, academic, community pharmacist), and, for students, year of study.

Questions 7 through 19 asked about 13 clusters of competences with a total of 68 competences. Questions in clusters 7 through 11 were concerned with personal competences and in clusters 12 through 19 with patient care competences (Appendix Table A1).

Respondents were asked to rank the proposals for competences with a 4-point Likert scale (table 2).

**Table 2:** Ranking of competences.

Rank	Significance	Explanation
1	Not important	Can be ignored
2	Quite important	Valuable but not obligatory
3	Very important	Obligatory, with exceptions depending upon field of pharmacy practice
4	Essential	Obligatory

There was also a “cannot rank” possibility as well as the possibility of leaving the answer blank; these numbers were pooled.

Results are presented in the form of “scores”: score = (frequency rank 3 + frequency rank 4) as % of total frequency. This calculation is based on that used by the MEDINE consortium [11] that studied the ranking of competences for medical practice by academics and medical students. Scores were used for descriptive purposes only.

Leik ordinal consensus, an indication of the ordinal dispersion characterization of the intra-group frequencies [12] was calculated using an in-house excel spreadsheet. Responses for Leik ordinal consensus were graded as they were in the MEDINE study:

- < 0.2 poor,
- 0.21 – 0.4 fair,
- 0.41 – 0.6 moderate,
- 0.61 – 0.8 substantial,
- > 0.81 good.

Data for the three groups were analyzed at three levels: overall, cluster of competences, and individual competences. Data comparing first and fifth year students were analyzed at the competence level. Data comparing scores from five countries with an arbitrarily chosen number of respondents ≥ 20 (see Table 1), with the nature or the content of the pharmacy course was also analyzed at the competence level only. The nature of the course was defined on the basis of the hours spent on medicinal and chemical sciences [13] and given as an index “medicinal/chemical score” = (% of total course hours spent on medicinal subjects /% of total course hours spent on chemical subjects). Medicinal subjects included pharmacology, therapeutics, *etc.*, and chemical subjects included analytical chemistry, organic chemistry, *etc.* The full list of subjects in each is given in the paper on heterogeneity on pharmacy education in Europe cited previously. Data were also compared to the course content: the relative amounts of time spent on lectures and on traineeship (data from the PHARMINE study).

The significance of differences between the results for ranking by groups was established using the chi-square test on the distribution of frequencies for the four ranks. A significance level of *p* < 0.05 was used (chi-square for three degrees of freedom (4 ranks − 1) = 7.81; *ns* = not significant).

Data is presented mainly as star plots. Star plots are suited to display multivariate ordinal observations with an arbitrary number of variables. This representation is suitable for the study of clusters as well as for detecting outliers [14].

All statistical tests were performed using GraphPad software [15].

## 3. Results and Discussion

The first level of analysis was the overall analysis of the pooled results (*n* = 68 competences). In Table 3 is given the distribution of rankings. For all three groups the response rate was high with only 6.9% to 11.7% unable to reply. This suggests that all groups of respondents considered they were sufficiently informed to reply to the questions asked.

**Table 3:** Overall distribution (over 68 competences) of rankings by students, community pharmacists, and academics.

	Students		Community Pharmacists		Academics	
Number of respondents	370		258		241	
Theoretical total number of replies	25,160 (= 370 × 68)		17,544 (= 258 × 68)		16,388 (= 241 × 68)	
Replies by rank	Frequency	%	Frequency	%	Frequency	%
4	8428	33.5	6643	37.9	5821	35.5
3	8967	35.6	6002	34.2	6005	36.6
2	4278	17.0	3076	17.5	2982	18.2
1	531	2.1	608	3.5	366	2.2
Cannot rank + blanks	619	11.7	1215	6.9	1214	7.4
Score (%)	77.4		78.3		77.9	
Leik ordinal consensus	0.59		0.55		0.58	

Scores for the three groups were similar and within the range of means of 77.4% to 78.3% showing that almost 80% of the competences proposed were considered “obligatory for practice” by all.

Values for Leik’s ordinal consensus were similar (0.55–0.59) and at the top end of the “moderate” grade (0.41–0.6). It should be noted, however, that this particular Leik analysis confounds groups and competences. Thus ordinal consensus may be moderate because there are differences amongst the groups and/or amongst the competences. Albeit as judged from the Leik ordinal consensus values, dispersion was relatively low. This suggests that groups were relatively homogeneous and there were no subgroups with responses significantly different from the others. Similar values for ordinal consensus were reported by the MEDINE consortium when they evaluated the ranking of competences for medical doctors. Their respondent population consisted of two-thirds academics delivering undergraduate medical education, and 28% medical students (see MEDINE citations above).

**Figure 1:**
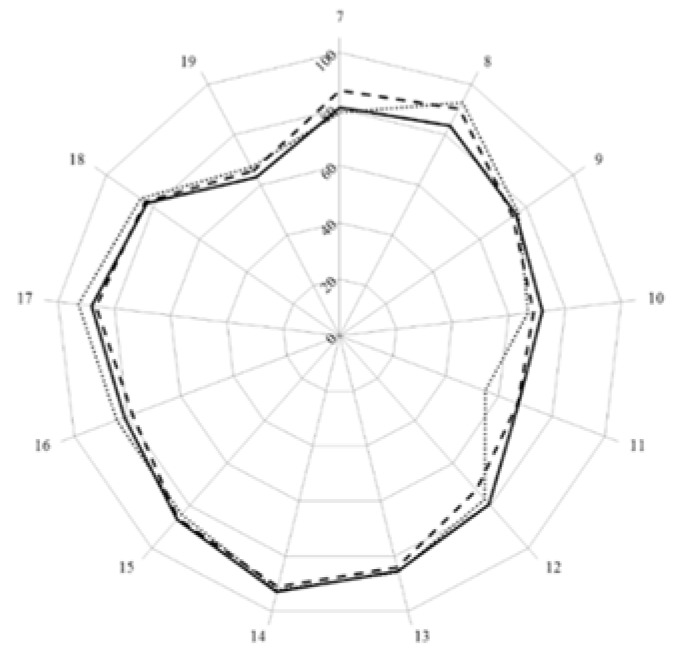
Scores (central vertical axe, 0%–100%) for the 13 (numbered 7 through 19) clusters of competences of students (full line), academics (dashed line), and community pharmacists (dotted line).

The second level of analysis was based on the grouping of competences into clusters representing the major domains of practice. In Figure 1 are given the scores for the 13 clusters of competences (numbered 7 through 19).

Scores for most clusters were 80% or above. Scores were lower than 80% for clusters of personal competences especially those for cluster 11 that dealt with industrial pharmacy. In this case, students had similar scores to academics (chi-square: 2.85, *ns*) and scored well above community pharmacists (chi-square: 89.04, *p* < 0.05). Students scored lower than academics for personal competence clusters 7 and 8, and lower than community pharmacists for cluster 8. Scores were also lower for cluster 19 (evaluation of outcomes) with no difference between students and academics (chi-square: 1.79, *ns*) or community pharmacists (chi-square: 3.19, *ns*).

**Figure 2:**
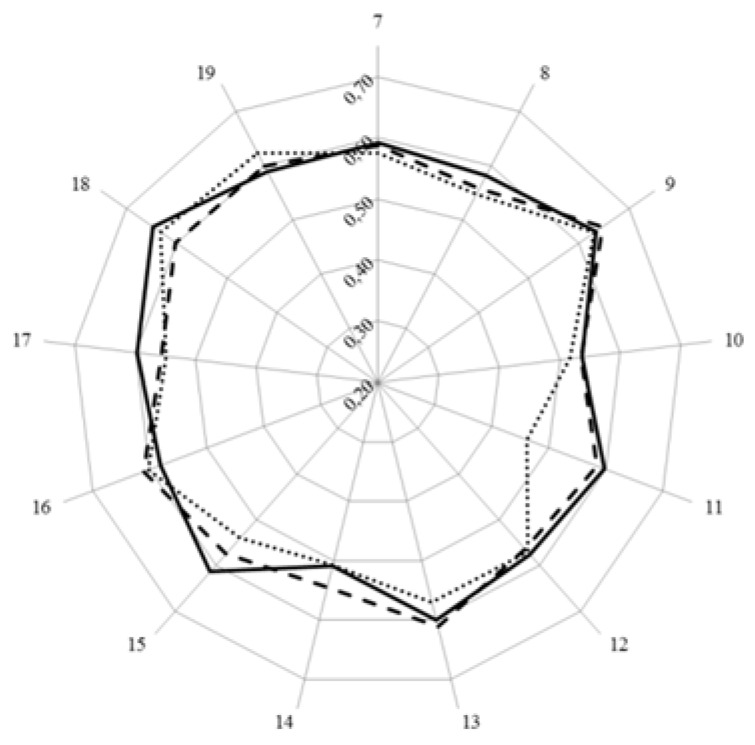
Leik’s ordinal consensus (central vertical axe, 0.2–0.7) for the 13 clusters of competences of students (full line), academics (dashed line), and community pharmacists (dotted line).

In Figure 2 are given the values for Leik’s ordinal consensus for the 13 clusters of competences (numbered 7 through 19).

For most clusters, ordinal consensus was at the top end of the 0.41–0.60 “moderate” category. Students (and academics) generally showed higher values than community pharmacists and this was especially true for cluster 11 which community pharmacists scored low (Figure 1) and showed a low ordinal consensus (Figure 2). This suggests that the low score for cluster 11 was not shared by all community pharmacists.

**Figure 3:**
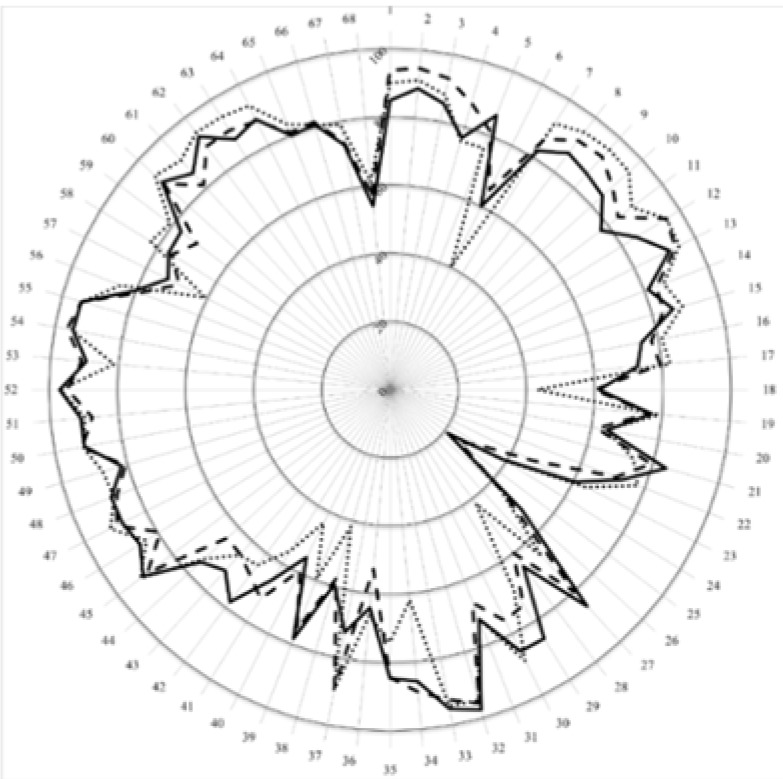
Scores (central vertical axe, 0%–100%) for the 68 competences of students (full line), academics (dashed line), and community pharmacists (dotted line).

The third level of analysis was at the level of competences. In Figure 3 are given the scores for the 68 competences (numbered 1 through 68 on the circumference). This figure shows that more detail amongst the groups is revealed by analysis at this third, competence level.

Significant differences between students and community pharmacists (Appendix Table A1) were seen in cluster 8 “personal competences 8–12: values” covering aspects such as contact, confidentiality, responsibility and ethics for which student scores were lower than those of community pharmacists. This was also seen but to lesser extent in the comparison between students and academics. Student scores for quality aspects of drug production and testing were higher than those of community pharmacists—cluster 11 (industrial pharmacy, competences 38–42) and competence 57 in cluster 15 “ability to manufacture medicinal products that are not commercially available”. It may be worthwhile in the future to consider focus groups in order to ascertain when and why do community pharmacists change their perspective on those competences (38–42) related to industrial pharmacy. Differences between students and academics were seen in cluster 7 “personal competences 1–7: learning and knowledge”; competences 1, 3, and 4 dealing with ability to learn independently and critical appraisal of relevant knowledge were scored lower by students.

Although competence 6 dealing with research issues was scored low by students (and by academics) the score was significantly higher than that of community pharmacists. This lack of recognition that pharmacy is a research-based discipline is paralleled by the lack of a substantial link between biomedical research and medical education and practice as described in the MEDINE study [16]. In the latter paper, Van Schravendijk and his MEDINE colleagues suggested ways of strengthening this link by bibliographic research and thesis work during pre-graduate study.

Such tools do exist in many pharmacy departments. In some cases this “science” aspect is taken even further with traineeships based on participation in clinical research topics in community and hospital pharmacy, and in pharmaceutical research and development in industrial settings. Further efforts are needed to promote such activities at a wider postgraduate level. Emphasis on such aspects in continuing professional development could help maintain the research-based nature of pharmacy.

Globally, the ranking by students, academics, and community pharmacists were similar. Patient care competences were ranked similarly by students and community pharmacists suggesting, importantly, that students have a good conception of their future job responsibilities and practice. Because there were few differences between academics and community pharmacists, it is also important to notice that academics have a good conception of the activity in community pharmacy. This critical nature of the “type of patient care provided by pharmacists” has been emphasized following evaluation of competences for pharmacists on a world-wide basis [17].

**Figure 4:**
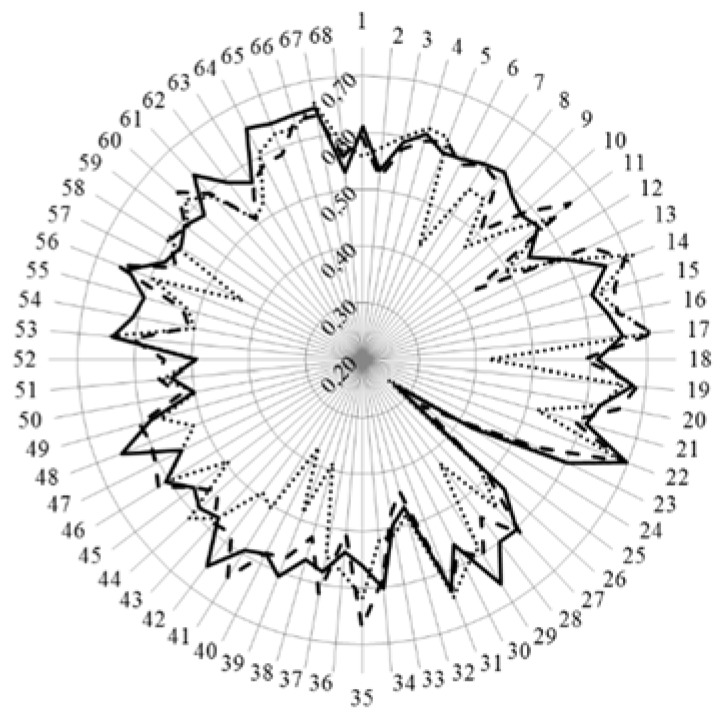
Leik’s ordinal consensus (central vertical axe, 0.2–0.7) for the 68 competences of students (full line), academics (dashed line), and community pharmacists (dotted line).

In Figure 4 are given the values for Leik’s ordinal consensus for the 13 clusters of competences (numbered 7 through 19).

For many competences ordinal consensus was lower in community pharmacists than in both students and academics. Ordinal consensus was low for all groups for “competences” 24 “biology” and 25 “physics”. These are, however, “subjects” not “competences”.

**Figure 5:**
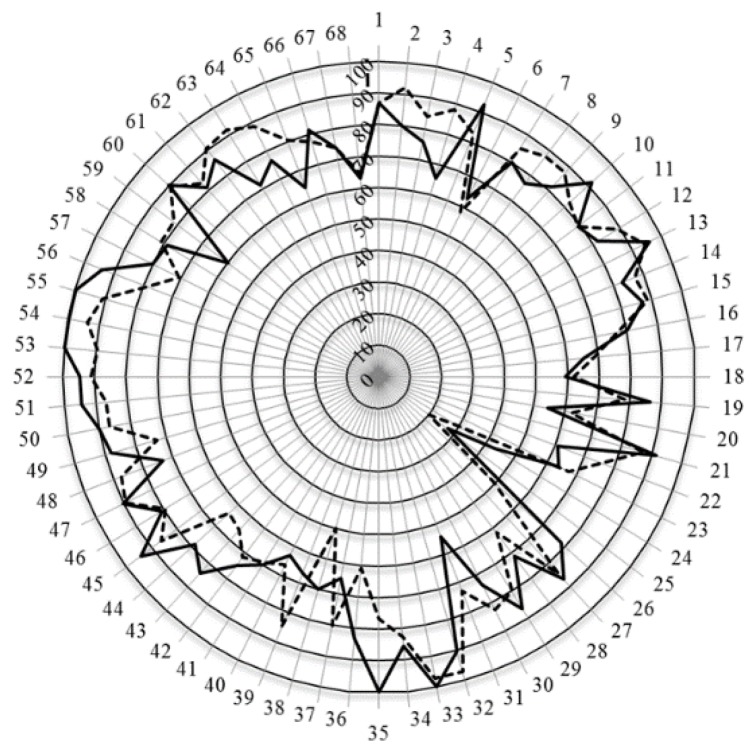
Ranking scores (central vertical axe, 0%–100%) for the 68 competences (on the circumference) by first (full line) and fifth (dotted line) year students.

Figure 5 shows the ranking scores for first (*n* = 30) and fifth (*n* = 77) students. Competences 24, 25, 26, 35, 36, 38, and 43 decreased in ranking from the first to the fifth year, whereas 4, 22, 31, 37, 39, 59, 63, and 65 increased.

The evolution of ranking throughout the pharmacy degree course, reflected by the changes in ranking between first and fifth year students, involved mainly personal values and subject areas. Ranks were fifth year > first year for competences 4, 22, 31, 37, and 39, and first year > fifth year for competences 24, 25, 26, 35, 36, 38, and 43. Three patient care competences increased in ranking throughout studies and these were 59 “provision of appropriate lifestyle advice on smoking, obesity, *etc.*”, 63 “provision of informed support for patients in selection and use of non-prescription medicines for minor ailments (e.g., cough remedies)”, and 65 “ability to monitor and report to all concerned in a timely manner, and in accordance with current regulatory guidelines on Good Pharmacovigilance Practices (GVPs), Adverse Drug Events and Reactions (ADEs and ADRs)”. This may be linked to the increased awareness of advanced students of their role as an advisor on health matters, especially so once they have undergone their traineeship in their final year.

**Figure 6:**
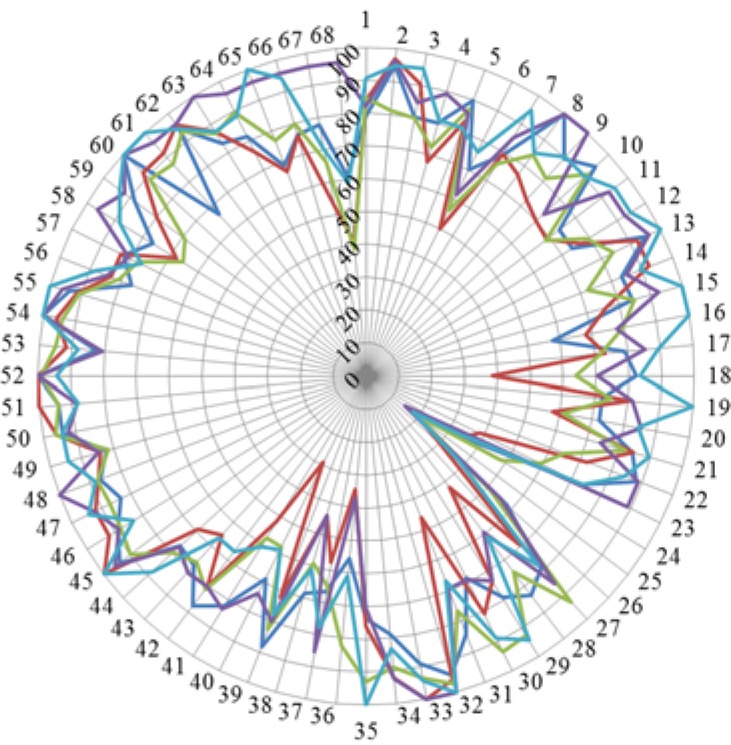
Rankings for five countries in which the respondent number was ≥ 20. Belgium: dark blue; Czech Republic: red; Germany: green; Portugal: mauve; Romania: light blue.

One final aspect concerns possible differences in results overall between participants from different countries. In Figure 6 are given the rankings for five countries in which the respondent number was ≥ 20.

Although the overall patterns of the rankings showed no large discrepancies amongst the different countries, there were significant differences in the mean scores (Table 4) with the Czech Republic giving the lowest score (73%) and Romania the highest (84%).

**Table 4:** Mean scores for five countries shown in Figure 6 together with data for course nature (= time spent on traineeship/time spent on lectures) and course content (= time spent on medicinal subjects/time spent on chemical subjects). Data from Atkinson (2014) and PHARMINE (2014) cited previously.

Country	Mean Ranking Score (%, *n* = 68 Competences)	Course Nature	Course Content
		Time Spent on Traineeship/Time Spent on Lectures	Time Spent on Medicinal Subjects/Time Spent on Chemical Subjects
Belgium	80	0.75	1.13
Czech Republic	73	0.76	1.12
Germany	78	1.25	0.71
Portugal	83	0.84	1.64
Romania	84	0.66	0.95

Although there are substantial differences in course nature and content this was not reflected in mean scores (Table 3) or in the patterns of the rankings (Figure 6). Some differences were observed: for example, students of the Czech Republic gave low scores for competence 18 (ability to design and manage the development processes in the production of medicines) and competence 40 (current knowledge of European directives on qualified persons (QPs)), but this was not observed for Belgium which has a similar course nature and content.

## 4. Conclusions

To our knowledge, this is the first study in which students in a sectoral profession are asked to rank the relative importance of competences for practice in their future professional lives. Globally, their perception of the relative importance of competences is similar to that of practicing community pharmacists especially in the area of patient care competences.

Given the growing interest in competence-based educational reforms in several areas of the world, it would be useful to do studies similar to this one in various areas worldwide in order to see whether student perceptions are equally advanced in all areas. This could be done through European-funded programs such as Erasmus+ [18] and would be one way of increasing awareness of and developing competence-based education in other regions. The results of such studies could be linked to the education framework used to train future pharmacists in those countries.

A proviso to this study is that it concentrates on community pharmacy practice. Whilst 70%–80% of pharmacists work in a community pharmacy in Europe (data from PHARMINE), many work in other areas such as hospital and industrial pharmacy. As education for jobs in the latter areas differs substantially amongst European countries, and the options for hospital and industrial pharmacy courses and training occur late in the courses it proved impossible to do a study similar to this in the specific areas of hospital or industrial pharmacy.

Another proviso is that, given the diversity of country of origin, it would have been useful to know something about the level of English proficiency for cohorts from different counties as the survey was delivered in English and if proficiency in English had any impact on the results. It would have been interesting to have information on the English proficiency, via for example, scores in the “Test of English as a Foreign Language (TOEFL)” examination [19]. However this is not uniformly applied in Europe and not even uniformly applied within each member state. We can comment that students ranked competence 21 “ability to communicate in English” very highly (score 85) suggesting that they at least recognized the necessity to understand English. It should be noted that the consortium strived to make the questions understandable to non-native English speakers.

Furthermore, when asked to rank subject areas listed in the European Directive many were ranked as “not important/can be ignored”. These are not competences [20] as such but components of competences (see Miller’s triangle, below). They were included in the questionnaire because they are cited in the European directive on the sectoral profession of pharmacy [21].

**Figure 7:**
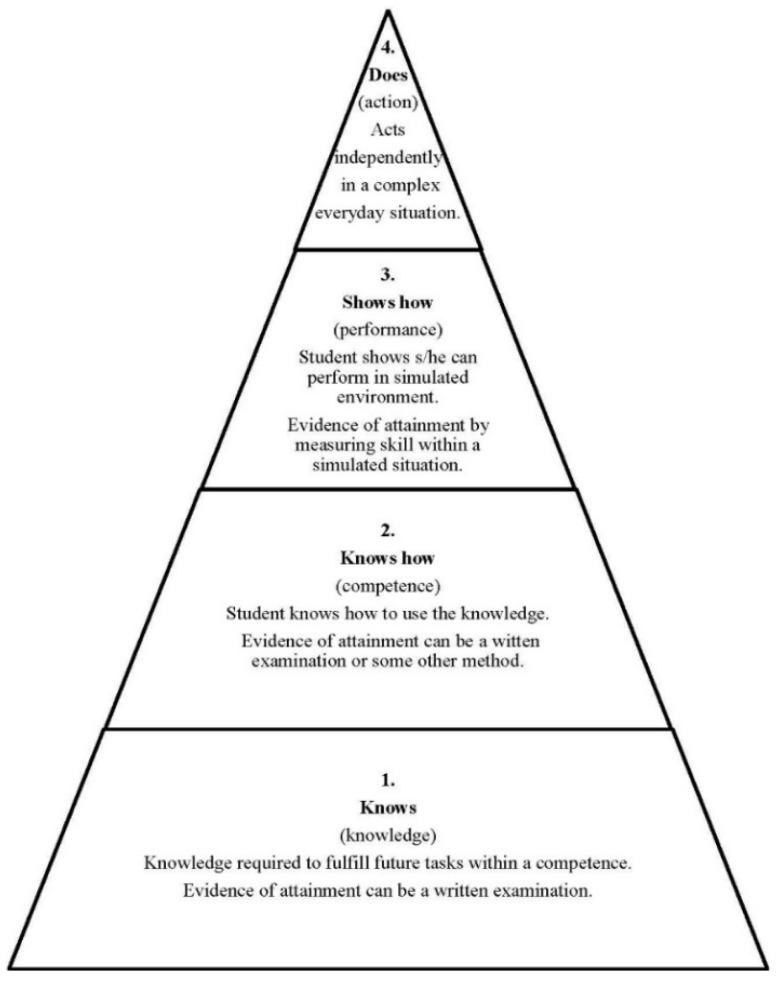
Miller’s triangle.

The final question to be discussed relates to how a competence framework could be incorporated into pharmacy education. This will be discussed in relation to Miller’s triangle that describes a conceptual, pyramidal model of the various facets of competence, in his case applied to clinical practice [22] (Figure 7).

The studies cited in the introduction on the use of competency frameworks for the improvement of performance are mostly concerned with the fourth level of this triangle—“does/action” whereas in this paper we are equally concerned with the other three levels, especially the first two “knows and knows how”. Given the low scores of most of the subject areas included in cluster 10, it would appear that students (as well as academics and community pharmacist practitioners) do not fully grasp the importance of some subjects as building blocks of the competences required for practice. This also outlines the fact that the simple inclusion of subject areas within a European regulatory directive is not a fully sufficient way to ensure the teaching and acquirement of competences for practice. One of the functions or uses of the PHAR-QA framework could be therefore to provide a road-book for the development of integrated, coordinated courses that groups several subject areas under a broad competence heading. The way in which this is to be carried out need not be harmonized or imposed by European directives or other legislative means but could be left to the wisdom of individual faculties to find solutions. Another function of the PHAR-QA competence framework could be as a basis of accreditation at level 2 of the Miller’s triangle allowing a more realistic evaluation of a student based on his/her competence to synthesise the different subject areas into a comprehensive understanding of different competences. This entails a switch in evaluation from normative procedures that rank candidates with arbitrary cut-off points to competence-referenced testing that evaluates whether a candidate is (or is not) ready to pass onto a higher level. The way in which this is developed would again be up to individual faculties. It should be noted that changing the method of evaluation is often a good beginning for a change in courses.

The PHAR-QA framework could also be used at the third level in the performance testing of students. Here one could also use the patient substitutes (or “standardized” patients as described by Miller cited previously). Such “patients” would present students with elements such as symptoms, prescriptions, *etc.* calling upon their competences to solve problems related to drug interactions, for example. How this would be constructed and run would again by up to the individual faculty. It should also be noticed that a change to a competency-based assessment would logically abandon a fixed time traineeship ship at level 3, as is specified by the EU directive and adopted by all EU countries at the present time, and its replacement by a system whereby the student would remain at level 3 in traineeship until s/he has shown to be competent enough to pass to level 1 and exercise his/her profession [23]. Whilst not going to such an extreme, it can be acknowledged that there is a (sometimes large) gap between levels 1 through 3 and level 4 or between the more theoretical academic approach and the real-life work situation. This could be addressed in junior pharmacists using the PHAR-QA framework as a basis for work-based self-directed learning and case-based assignments set and monitored by a senior colleague (see the Rutter, *et al.* reference cited in the introduction).

## Appendix

**Table A1:** Ranking scores for competences by groups (students, academics, community pharmacists). Note that the numbering of the clusters of competences starts at 7, *i.e.*, after the 6 questions on profile of respondent). N: number of competence. Stud.: students. Acad.: academics. Comm.: community pharmacists. Chi: chi-square. *vs.*: *versus*.

	N	Competence	Stud.	Acad.	Chi Stud. *vs.* Acad.	Comm.	Chi Stud. *vs*. Comm.
Cluster 7. Personal competences: learning and knowledge.	1	Ability to identify learning needs and to learn independently (including continuous professional development (CPD)).	84.5	93.7	15.7	89.8	13.1
	2	Analysis: ability to apply logic to problem solving, evaluating pros and cons and following up on the solution found.	88.8	94.5	7.5	91.1	3.6
	3	Synthesis: capacity to gather and critically appraise relevant knowledge and to summarise the key points.	85.1	92.8	10.8	87.9	4.0
	4	Capacity to evaluate scientific data in line with current scientific and technological knowledge.	76.5	87.3	18.5	75.8	0.4
	5	Ability to interpret preclinical and clinical evidence-based medical science and apply the knowledge to pharmaceutical practice.	86.0	81.2	5.2	75.9	17.3
	6	Ability to design and conduct research using appropriate methodology.	60.6	65.4	4.9	40.2	34.3
	7	Ability to maintain current knowledge of relevant legislation and codes of pharmacy practice.	81.7	86.3	3.3	91.7	25.7
Cluster 8. Personal competences: values.	8	Demonstrate a professional approach to tasks and human relations.	86.6	91.5	7.7	94.5	23.3
	9	Demonstrate the ability to maintain confidentiality.	85.4	92.3	22.8	95.3	50.6
	10	Take full personal responsibility for patient care and other aspects of one’s practice.	84.4	88.3	3.2	94.8	24.9
	11	Inspire the confidence of others in one’s actions and advice.	77.8	83.8	8.9	88.8	13.0
	12	Demonstrate high ethical standards.	85.3	95.3	43.4	95.2	24.6
Cluster 9. Personal competences: communication and organisational skills.	13	Effective communication skills (both orally and written).	91.2	93.5	3.9	94.8	4.0
	14	Effective use of information technology.	81.1	83.8	1.4	86.1	3.8
	15	Ability to work effectively as part of a tea.	86.4	83.3	6.1	89.2	1.1
	16	Ability to identify and implement legal and professional requirements relating to employment (e.g., for pharmacy technicians) and to safety in the workplace.	74.8	77.9	1.9	81.0	4.5
	17	Ability to contribute to the learning and training of staff.	73.5	79.6	6.6	82.5	6.6
	18	Ability to design and manage the development processes in the production of medicines.	61.2	60.0	0.8	43.2	38.0
	19	Ability to identify and manage risk and quality of service issues.	77.5	76.1	4.0	79.2	2.3
	20	Ability to identify the need for new services.	65.0	61.8	7.7	64.5	1.2
	21	Ability to communicate in English and/or locally relevant languages.	84.5	79.6	2.3	74.1	16.3
	22	Ability to evaluate issues related to quality of service.	73.0	71.0	3.5	77.9	7.4
	23	Ability to negotiate, understand a business environment and develop entrepreneurship.	62.2	46.4	15.6	64.1	2.0
Cluster 10. Personal competences: knowledge of different areas of the science of medicines.	24	Plant and animal biology.	38.8	31.1	5.1	39.3	1.0
	25	Physics.	20.9	25.6	2.3	21.7	0.8
	26	General and inorganic chemistry	53.0	45.6	3.3	43.9	5.3
	27	Organic and medicinal/pharmaceutical chemistry.	86.3	80.2	10.8	66.0	37.0
	28	Analytical chemistry	65.8	60.0	3.0	41.9	46.9
	29	General and applied biochemistry (medicinal and clinical).	85.4	74.2	10.8	68.8	22.6
	30	Anatomy and physiology; medical terminology.	85.2	75.8	11.2	88.7	3.3
	31	Microbiology.	72.2	67.0	3.3	72.2	1.5
	32	Pharmacology including pharmacokinetics.	97.5	95.6	3.7	94.7	3.0
	33	Pharmacotherapy and pharmaco-epidemiology.	95.3	92.5	3.1	94.3	2.2
	34	Pharmaceutical technology including analyses of medicinal products.	86.9	89.0	1.4	62.0	50.8
	35	Toxicology.	85.0	84.4	17.3	74.0	27.7
	36	Pharmacognosy.	65.9	52.9	11.3	66.5	2.1
	37	Legislation and professional ethics.	71.7	88.8	26.8	89.5	44.2
Cluster 11. Personal competences: understanding of industrial pharmacy.	38	Current knowledge of design, synthesis, isolation, characterisation and biological evaluation of active substances.	59.9	57.5	1.9	41.7	34.2
	39	Current knowledge of good manufacturing practice (GMP) and of good laboratory practice (GLP).	79.2	75.4	1.6	59.4	29.8
	40	Current knowledge of European directives on qualified persons (QPs).	55.3	59.2	1.8	43.7	39.9
	41	Current knowledge of drug registration, licensing and marketing.	65.7	72.1	4.6	55.7	11.9
	42	Current knowledge of good clinical practice (GCP).	78.1	68.2	9.1	64.5	23.8
Cluster 12. Patient care competences: patient consultation and assessment.	43	Ability to perform and interpret medical laboratory tests.	72.0	65.3	5.9	65.5	6.0
	44	Ability to perform appropriate diagnostic or physiological tests to inform clinical decision making e.g., measurement of blood pressure.	76.1	64.5	17.3	73.6	7.8
	45	Ability to recognise when referral to another member of the healthcare team is needed because a potential clinical problem is identified (pharmaceutical, medical, psychological or social).	91.7	89.1	2.2	91.7	9.5
Cluster 13. Patient care competences: need for drug treatment.	46	Retrieval and interpretation of relevant information on the patient’s clinical background.	85.6	79.3	8.4	84.0	0.7
	47	Retrieval and interpretation of an accurate and comprehensive drug history if and when required.	87.6	89.4	5.1	91.5	2.3
	48	Identification of non-adherence and implementation of appropriate patient intervention.	87.1	85.8	6.1	86.8	24.5
	49	Ability to advise to physicians and—in some cases—prescribe medication.	81.9	80.7	2.5	87.6	5.3
Cluster 14. Patient care competences: drug interactions.	50	Identification, understanding and prioritisation of drug-drug interactions at a molecular level (e.g., use of codeine with paracetamol).	91.4	91.8	1.1	91.6	0.6
	51	Identification, understanding, and prioritisation of drug-patient interactions, including those that preclude or require the use of a specific drug (e.g., trastuzumab for treatment of breast cancer in women with HER2 overexpression).	91.4	87.7	4.4	89.7	5.0
	52	Identification, understanding, and prioritisation of drug-disease interactions (e.g., NSAIDs in heart failure).	97.0	94.5	8.9	96.6	2.7
Cluster 15. Patient care competences: provision of drug product.	53	Familiarity with the bio-pharmaceutical, pharmacodynamic and pharmacokinetic activity of a substance in the body.	89.3	90.8	3.5	81.2	11.6
	54	Supply of appropriate medicines taking into account dose, correct formulation, concentration, administration route and timing.	94.3	96.3	16.3	94.9	18.0
	55	Critical evaluation of the prescription to ensure that it is clinically appropriate and legal.	93.9	94.1	6.6	94.0	11.1
	56	Familiarity with the supply chain of medicines and the ability to ensure timely flow of drug products to the patient.	81.6	78.6	4.5	84.6	11.3
	57	Ability to manufacture medicinal products that are not commercially available.	74.1	69.0	1.5	60.5	21.2
Cluster 16. Patient care competences: patient education.	58	Promotion of public health in collaboration with other actors in the healthcare system.	75.8	75.1	1.1	82.6	5.9
	59	Provision of appropriate lifestyle advice on smoking, obesity, *etc*.	76.9	71.0	3.8	80.9	4.7
	60	Provision of appropriate advice on resistance to antibiotics and similar public health issues.	90.3	89.4	5.2	93.1	3.6
Cluster 17. Patient care competences: provision of information and service.	61	Ability to use effective consultations to identify the patient’s need for information.	85.6	81.1	3.1	90.9	11.1
	62	Provision of accurate and appropriate information on prescription medicines.	92.7	89.3	8.0	94.4	11.0
	63	Provision of informed support for patients in selection and use of non-prescription medicines for minor ailments (e.g., cough remedies).	85.7	89.4	1.7	94.0	14.4
Cluster 18. Patient care competences: monitoring of drug therapy.	64	Identification and prioritisation of problems in the management of medicines in a timely manner and with sufficient efficacy to ensure patient safety.	88.5	87.9	8.2	93.0	8.7
	65	Ability to monitor and report to all concerned in a timely manner, and in accordance with current regulatory guidelines on Good Pharmacovigilance Practices (GVPs), Adverse Drug Events and Reactions (ADEs and ADRs).	79.8	80.9	5.0	83.4	3.3
	66	Undertaking of a critical evaluation of prescribed medicines to confirm that current clinical guidelines are appropriately applied.	80.7	81.6	0.3	80.6	4.5
Cluster 19. Patient care competences: evaluation of outcomes.	67	Assessment of outcomes on the monitoring of patient care and follow-up interventions.	73.3	73.7	0.5	79.0	4.4
	68	Evaluation of cost effectiveness of treatment.	53.3	57.7	2.1	61.2	4.8

Chi-square, d. f. 3, *p* = 0.05: 7.8. The chi-square test was performed on the frequencies of rankings.
